# Survival Risk Scores for Real-Life Relapsed/Refractory Multiple Myeloma Patients Receiving Elotuzumab or Carfilzomib In Combination With Lenalidomide and Dexamethasone as Salvage Therapy: Analysis of 919 Cases Outside Clinical Trials

**DOI:** 10.3389/fonc.2022.890376

**Published:** 2022-07-18

**Authors:** Fortunato Morabito, Elena Zamagni, Concetta Conticello, Vincenzo Pavone, Salvatore Palmieri, Sara Bringhen, Monica Galli, Silvia Mangiacavalli, Daniele Derudas, Elena Rossi, Roberto Ria, Lucio Catalano, Paola Tacchetti, Giuseppe Mele, Iolanda Donatella Vincelli, Enrica Antonia Martino, Ernesto Vigna, Antonella Bruzzese, Francesco Mendicino, Cirino Botta, Anna Mele, Lucia Pantani, Serena Rocchi, Bruno Garibaldi, Nicola Cascavilla, Stelvio Ballanti, Giovanni Tripepi, Ferdinando Frigeri, Antonetta Pia Falcone, Clotilde Cangialosi, Giovanni Reddiconto, Giuliana Farina, Marialucia Barone, Ilaria Rizzello, Enrico Iaccino, Selena Mimmi, Paola Curci, Barbara Gamberi, Pellegrino Musto, Valerio De Stefano, Maurizio Musso, Maria Teresa Petrucci, Massimo Offidani, Francesco Di Raimondo, Mario Boccadoro, Michele Cavo, Antonino Neri, Massimo Gentile

**Affiliations:** ^1^ Biotechnology Research Unit, AO of Cosenza, Cosenza, Italy; ^2^ Hematology and Bone Marrow Transplant Unit, Hemato-Oncology Department, Augusta Victoria Hospital, East Jerusalem, Israel; ^3^ IRCCS Azienda Ospedaliero-Universitaria di Bologna, Istituto di Ematologia “Seràgnoli”, Bologna, Italy; ^4^ Dipartimento di Medicina Specialistica, Diagnostica e Sperimentale, Università di Bologna, Bologna, Italy; ^5^ Division of Hematology, Azienda Policlinico-S. Marco, University of Catania, Catania, Italy; ^6^ Department of Hematology and Bone Marrow Transplant, Hospital Card. G. Panico, Tricase, Italy; ^7^ Hematology Unit, Ospedale Cardarelli, Napoli, Italy; ^8^ Division of Hematology, AOU Città della Salute e della Scienza di Torino, University of Torino, Torino, Italy; ^9^ Hematology and Bone Marrow Transplant Unit, Azienda Socio-Sanitaria Territoriale-Papa Giovanni XXIII, Bergamo, Italy; ^10^ Hematology Division, Department of Hematology-Oncology, IRCCS Fondazione Policlinico San Matteo, Pavia, Italy; ^11^ Department of Hematology, Businco Hospital, Cagliari, Italy; ^12^ Istituto di Ematologia, Università Cattolica, Fondazione Policlinico Gemelli IRCCS, Roma, Italy; ^13^ Department of Biomedical Science, Internal Medicine “G. Baccelli”, Policlinico, University of Bari “Aldo Moro” Medical School, Bari, Italy; ^14^ Hematology, AUOP “Federico II”, Naples, Italy; ^15^ Department of Hematology, Hospital Perrino, Brindisi, Italy; ^16^ Hematology Unit, Department of Hemato-Oncology and Radiotherapy, Great Metropolitan Hospital “Bianchi-Melacrino-Morelli”, Reggio Calabria, Italy; ^17^ Department of Onco-Hematology, Hematology Unit AO of Cosenza, Cosenza, Italy; ^18^ Department of Hematology and Bone Marrow Transplant, IRCCS Casa Sollievo della Sofferenza, San Giovanni Rotondo, Italy; ^19^ Institute of Haematology and Stem Cell transplantation, Ospedale Santa Maria della Misericordia, University of Perugia, Perugia, Italy; ^20^ Department of Internal Medicine, Nephrology Center of National Research Institute of Biomedicine and Molecular Immunology, Reggio Calabria, Italy; ^21^ UOC Ematologia a Indirizzo Oncologico, AORN “Sant’Anna e San Sebastiano”, Caserta, Italy; ^22^ U.O.C. Ematologia A. O. Ospedali Riuniti Villa Sofia-Cervello, Palermo, Italy; ^23^ Department of Hematology, Hospital Vito Fazzi, Lecce, Italy; ^24^ Onco-Hematology, “Tortora” Hospital, Pagani, Italy; ^25^ Department of Experimental and Clinical Medicine, University “Magna Graecia” of Catanzaro, Catanzaro, Italy; ^26^ Department of Emergency and Organ Transplantation, “Aldo Moro” University School of Medicine and Unit of Hematology and Stem Cell Transplantation, AOUC Policlinico, Bari, Italy; ^27^ Division of Hematology, Azienda USL-IRCCS of Reggio Emilia, Reggio Emilia, Italy; ^28^ U.O.C. OncoEmatologia e TMO, Dipartimento Oncologico, Palermo, Italy; ^29^ Department of Cellular Biotechnologies and Hematology, Sapienza University of Rome, Roma, Italy; ^30^ Hematology Unit, AOU Ospedali Riuniti di Ancona, Ancona, Italy; ^31^ Scientific Directorate, Azienda USL-IRCCS of Reggio Emilia, Reggio Emilia, Italy

**Keywords:** multiple myeloma, prognosis, prognostic score, carfilzomib, elotuzumab, lenalidomide, survival, relapsed/refractory

## Abstract

The present study aimed to develop two survival risk scores (RS) for overall survival (OS, SRS*^KRd/EloRd^*) and progression-free survival (PFS, PRS*^KRd/EloRd^*) in 919 relapsed/refractory multiple myeloma (RRMM) patients who received carfilzomib, lenalidomide, and dexamethasone (KRd)/elotuzumab, lenalidomide, and dexamethasone (EloRd). The median OS was 35.4 months, with no significant difference between the KRd arm versus the EloRd arm. In the multivariate analysis, advanced ISS (HR = 1.31; *P* = 0.025), interval diagnosis–therapy (HR = 1.46; *P* = 0.001), number of previous lines of therapies (HR = 1.96; *P* < 0.0001), older age (HR = 1.72; *P* < 0.0001), and prior lenalidomide exposure (HR = 1.30; *P* = 0.026) remained independently associated with death. The median PFS was 20.3 months, with no difference between the two strategies. The multivariate model identified a significant progression/death risk increase for ISS III (HR = 1.37; *P* = 0.002), >3 previous lines of therapies (HR = 1.67; *P* < 0.0001), older age (HR = 1.64; *P* < 0.0001), and prior lenalidomide exposure (HR = 1.35; *P* = 0.003). Three risk SRS*^KRd/EloRd^* categories were generated: low-risk (134 cases, 16.5%), intermediate-risk (467 cases, 57.3%), and high-risk categories (213 cases, 26.2%). The 1- and 2-year OS probability rates were 92.3% and 83.8% for the low-risk (HR = 1, reference category), 81.1% and 60.6% (HR = 2.73; *P* < 0.0001) for the intermediate-risk, and 65.5% and 42.5% (HR = 4.91; *P* < 0.0001) for the high-risk groups, respectively. Notably, unlike the low-risk group, which did not cross the median timeline, the OS median values were 36.6 and 18.6 months for the intermediate- and high-risk cases, respectively. Similarly, three PRS*^KRd/EloRd^* risk categories were engendered. Based on such grouping, 338 (41.5%) cases were allocated in the low-, 248 (30.5%) in the intermediate-, and 228 (28.0%) in the high-risk groups. The 1- and 2-year PFS probability rates were 71.4% and 54.5% for the low-risk (HR = 1, reference category), 68.9% and 43.7% (HR = 1.95; *P* < 0.0001) for the intermediate-risk, and 48.0% and 27.1% (HR = 3.73; *P* < 0.0001) for the high-risk groups, respectively. The PFS median values were 29.0, 21.0, and 11.7 months for the low-, intermediate-, and high-risk cases. This analysis showed 2.7- and 4.9-fold increased risk of death for the intermediate- and high-risk cases treated with KRd/EloRd as salvage therapy. The combined progression/death risks of the two categories were increased 1.3- and 2.2-fold compared to the low-risk group. In conclusion, SRS*^KRd/EloRd^* and PRS*^KRd/EloRd^* may represent accessible and globally applicable models in daily clinical practice and ultimately represent a prognostic tool for RRMM patients who received KRd or EloRd.

## Introduction

With the introduction of novel agents in clinical practice, such as immunomodulatory drugs (IMIDs), proteasome inhibitors (PIs), and more recently monoclonal antibodies (MoAbs), the survival of multiple myeloma (MM) patients dramatically improved in the last decade (1). However, despite unquestionable progress, patients ultimately relapse, possibly developing cross-drug resistance, with a high chance of a reduced response duration to successive lines of therapies (2–6). Nevertheless, through the accessibility of various distinctive classes of approved drugs, differently combined in doublet, triplet, or even quadruplet regimens and integrated, when appropriate, with autologous stem-cell transplantation (ASCT) procedure (7–10), myeloma treatment has changed drastically. The regimen selection mainly depends on many patient- (i.e., age, fitness status, number of exposure and refractoriness to previous therapies) (11) and neoplastic cell- (i.e., cytogenetics) (12) associated characteristics.

The three-drug combinations are definitively superior in improving outcome indicators compared to doublet treatments, hence representing the new standard of care for relapsed/refractory multiple myeloma (RRMM) (13, 14). Moreover, the peculiar mechanism of action of IMiDs, i.e., their immunomodulatory effects through the induction of NK cell activation and boosted ADCC activity (15), indicated IMiD-based protocols as intriguing backbone doublets to be integrated, in triplet regimens, with the second-class proteasome inhibitor carfilzomib (KRd) (16, 17), or with two primary monoclonal antibodies, recognizing the signaling lymphocytic activation molecule F7 (SLAMF7) (elotuzumab, EloRd) (18, 19) or CD38 (daratumumab) (20, 21).

Due to restrictive inclusion and exclusion criteria, the clinical features of cases registered in clinical trials generally do not fully match those of patients treated in clinical practice. Indeed, research in harmonizing clinical trial results with the outcomes highlighted in the real-world scenario is an appropriate scientific approach deserving additional investigation and constant updates. In this respect, the relatively extended follow-up of the two most popular triplets in the RRMM setting, i.e., KRd and EloRD, allowed us to analyze several real-life landscapes (22–27).

Although many prognostic scores to stratify newly diagnosed MM patients have been proposed, such as the Revised International Staging System (R-ISS) (12), which allows segregating cases according to serum markers (albumin, β2-microglobulin, lactate dehydrogenase) and cytogenetic abnormalities detected by fluorescence *in-situ* hybridization, there are few data in the literature regarding the prognostic tools applicable to RRMM patients exposed to specific treatments.

The present study aimed to develop two weighted, multivariate risk scores (RS) for overall survival (OS, SRS*^KRd/EloRd^*) and progression-free survival (PFS, PRS*^KRd/EloRd^*) by integrating several parameters in an independent real-life cohort of 919 RRMM patients who received KRd or EloRd salvage therapy outside of clinical trials.

## Materials and Methods

### Patients

For the aim of this retrospective analysis, the updated clinical data of five independent retrospective cohorts of RRMM patients partially included in previous papers (22–29), outside of clinical trials between December 2015 and December 2018, were collected. The five databases were merged into a single meta-database. Twenty other unpublished cases were also integrated. A Consolidated Standards Of Reporting Trials (CONSORT) diagram encompassing the enrollment phases of the real-world cases is depicted in **Supplementary Figure 1**.

EloRd and KRd patients were treated according to marketing approval (29). The refractoriness designates disease in patients who achieve a minor response (MR) or better and either become non-responsive while undergoing salvage therapy or who progress within 60 days of the last treatment (30).

The study was approved by institutional ethics committees according to the principles of the Declaration of Helsinki.

### Statistical Analysis

Data are expressed as absolute numbers and percentages or median and range.

Progression-free survival (PFS) was calculated from KRd/EloRd time to disease progression or death (event) or last follow-up (censoring). Overall survival (OS) was measured from the start of KRd or EloRd treatment until death from any cause. OS or PFS was censored at the last date of patient follow-up. The relationship between risk factors and the outcome variables was investigated by univariate and multiple Cox regression analyses. On univariate Cox regression analyses, the tested covariates for progression or death included age, gender, prior exposure to autologous stem cell transplant (ASCT) as well as to lenalidomide, lactate dehydrogenase (LDH), disease status at KRd/EloRd start, number of previous lines of therapies, and International Staging System (ISS) stage (31). All univariate correlates of progression/death or death for any cause were jointly introduced into the same multiple Cox regression model. Data were expressed as hazard ratio (HR), 95% confidence interval (CI), and *P*-value.

Time-to-event outcomes, i.e., PFS and OS, were calculated by the Kaplan–Meier method, and *P <*0.05 defined the statistical significance. The predictive cutoff value of age (optimal threshold) for discriminating patients who progressed or died or died for any cause from those without these outcomes was identified by the receiver operating characteristic (ROC) curve analysis. The prognostic value of the two risk prediction rules and other biomarkers/risk factors was assessed by calculating the area under the ROC curve (AUC).

We estimated differences in the relationship between individual independent risk factors and PFS or OS, utilizing the *_b_* Score as previously described (32) since literature data indicate that such an approach is superior to the risk ratio-based scoring system in predicting mortality (32). In detail, *_b_* Score was calculated by deriving a weight for each prognostic variable using each regression coefficient (*b*). The regression coefficients of the independent prognostic factors were preliminarily summed up. Then they were divided by this sum and multiplied by 100, thus deriving a weight for each predictive variable ranging from 0 (in unexposed patients) to a given percentage (in exposed individuals), the latter being proportional to the weight of each regression coefficient over the sum of the regression coefficients. These weights were summed up individually, thus deriving a score interpretable in a prognostic scale ranging from 0% (for patients unexposed to all risk factors) to 100% (for patients exposed to all risk factors). The agreement between the risk stratification provided by SRS*^KRd/EloRd^* and PRS*^KRd/EloRd^* was investigated by weighted kappa statistics. Kappa results are interpreted as follows: values ≤0 as indicating no agreement and 0.01–0.20 as none to slight, 0.21–0.40 as fair, 0.41–0.60 as moderate, 0.61–0.80 as substantial, and 0.81–1.00 as almost perfect agreement. *P*-value <0.05 was considered significant.

Data analysis was performed using STATA for Windows v.9, College Station, TX: StataCorp LLC and SPSS Statistics v.21, IBM, Chicago, Illinois.

## Results

### Patients’ Characteristics

Nine hundred and nineteen RRMM patients received treatment with KRd or EloRd in a real-world scenario. Patients’ characteristics, detected before the KRd/EloRd start, are depicted in **Table 1**. At KRd/EloRd start, the median age was 67 years (range 33–91). Half of the cases were women. ISS stratification information was available in 814 cases (88.6%); 237 cases (29.1%) were allocated in ISS stage III, 31.8% in ISS stage II, and 39.1% in ISS stage I. Many cytogenetic analyses were missing in this cohort, reflecting the non-routine attitude in performing this test in the real-world scenario. The available cases were 297 cases (32.5%), and less than one-quarter of them presented unfavorable cytogenetic abnormalities [i.e., t(4;14), t(14;16), or del(17p)]. Abnormal LDH serum level was detected in 407/786 available cases (51.8%). The median number of prior lines of therapy was 1 (range 1–11), with 53.9% of patients receiving KRd/EloRd after 1 line of therapy; 36.1% of the entire cohort was already exposed to lenalidomide (**Table 1**). Three hundred and forty cases received a previous ASCT. Finally, refractoriness to the last therapy was detected in 27% of the cases.

**Table 1:** Clinical features of 919 relapsed/refractory multiple myeloma (RRMM) patients treated with carfilzomib, lenalidomide, and dexamethasone (KRd) or elotuzumab, lenalidomide, and dexamethasone (EloRd) as salvage regimens in a real-life setting.

Age	
Median, years	67
Range	33–91
Gender	
Male, *n* (%)	459 (49.9)
Female, *n* (%)	460 (50.1)
International Stage System (ISS)	
I, *n* (%)	318 (39.1)
II, *n* (%)	259 (31.8)
III, *n* (%)	237 (29.1)
Missing, *n*	105
FISH analysis	
Standard risk, *n* (%)	229 (77.1)
High risk, *n* (%)	68 (22.9)
Missing, *n*	622
LDH	
Normal, *n* (%)	379 (48.2)
Abnormal, *n* (%)	407 (51.8)
Missing, *n*	133
Number of lines of previous therapy	
1 line	495 (53.9)
2 lines	205 (22.3)
3 lines	102 (11.1)
>3 lines	117 (12.7)
Previous exposure to lenalidomide	
No, *n* (%)	615 (66.9)
Yes, *n* (%)	304 (33.1)
Previous ASCT (autologous stem cell transplant)	
No, *n* (%)	529 (57.6)
Yes, *n* (%)	340 (42.4)
Disease status at KRd/EloRd start	
Relapse, *n* (%)	670 (72.9)
Refractory, *n* (%)	249 (27.1)

### Overall Survival

After a median follow-up of 18 months, 372 patients died. The median OS of the entire cohort was 35.4 months (95% CI 31.4–39.3), with no significant difference between the KRd arm versus the EloRd arm (**Supplementary Figure 2**). Univariate analyses showed that the interval diagnosis–therapy >3.5 years reduced the death risk by 23% (HR = 0.77, 95% CI 0.63–0.95, *P* = 0.015). Conversely, refractoriness status at KRd/EloRd start (HR = 1.25, 95% CI 1.01–1.55, *P* = 0.048), prior exposure to lenalidomide (HR = 1.25, 95% CI 1.02–1.54, *P* = 0.036), ISS III (HR = 1.48, 95% CI 1.18–1.86, *P* < 0.001), age >65.5 years as detected by ROC curve analysis (**Supplementary Figure 3**) (HR = 1.69, 95% CI 1.3–2.1, *P* < 0.001), and >3 previous lines of therapies (HR = 2.07, 95% CI 1.58–2.70, *P* < 0.001) were significantly associated with a higher risk to die. Previous ASCT was not significantly associated with OS [HR 1.1 (95% CI 0.9–1.36), *P* = 0.3].

When all variables showing a significant impact on OS were jointly introduced into the same multivariate model, fitted in 814 out of 919 patients (i.e., 88.6%), advanced ISS (HR = 1.31, 95% CI 1.03–1.65, *P* = 0.025), interval diagnosis–therapy (HR = 1.46, 95% CI 1.16–1.84, *P* = 0.001), number of previous lines of therapies (HR = 1.96, 95% CI 1.44–2.66, *P* < 0.001), age (HR = 1.72, 95% CI 1.36–2.18, *P* < 0.001), and prior lenalidomide exposure (HR = 1.30, 95% CI 1.03–1.65, *P* = 0.026) remained independently associated with death. Conversely, disease status at therapy start failed to maintain its independent prognostic role after the multiple data adjustment.

### Progression-Free Survival

The median PFS for the overall population was 20.3 months (95% CI 18.2–22.4) with no significant impact of the two therapeutic strategies (**Supplementary Figure 4**). At Cox univariate analysis for PFS, we analyzed all nine variables considered for OS. Four of them remained significantly associated with a higher risk of progression or death, i.e., prior exposure to lenalidomide (HR = 1.40, 95% CI 1.17–1.67, *P* < 0.001), ISS III (HR = 1.47, 95% CI 1.20–1.79, *P* < 0.001), age >65.9 years as identified by ROC curve analysis (**Supplementary Figure 5**) (HR = 1.55, 95% CI 1.29–1.87, *P* < 0.001), and >3 previous lines of therapies (HR = 2.10, 95% CI 1.67–2.67, *P* < 0.001) were significantly associated with a higher progression/death risk. Previous ASCT was not significantly associated with PFS [HR 1.07 (95% CI 0.9–1.28), *P* = 0.43].

In the multivariate analysis, all the variables mentioned above also maintained their independent association with death risk. Specifically, the model identified a significant increased progression/death risk for ISS III (HR = 1.37, 95% CI 1.12–1.67, *P* = 0.002), >3 previous lines of therapies (HR = 1.67, 95% CI 1.29–2.16, *P* < 0.0001), age >65.9 (HR = 1.64, 95% CI 1.34–1.99, *P* < 0.0001), and prior lenalidomide exposure (HR = 1.35, 95% CI 1.11–1.64, *P* = 0.003).

### Prognostic Survival Scoring Systems

**Figure 1:**
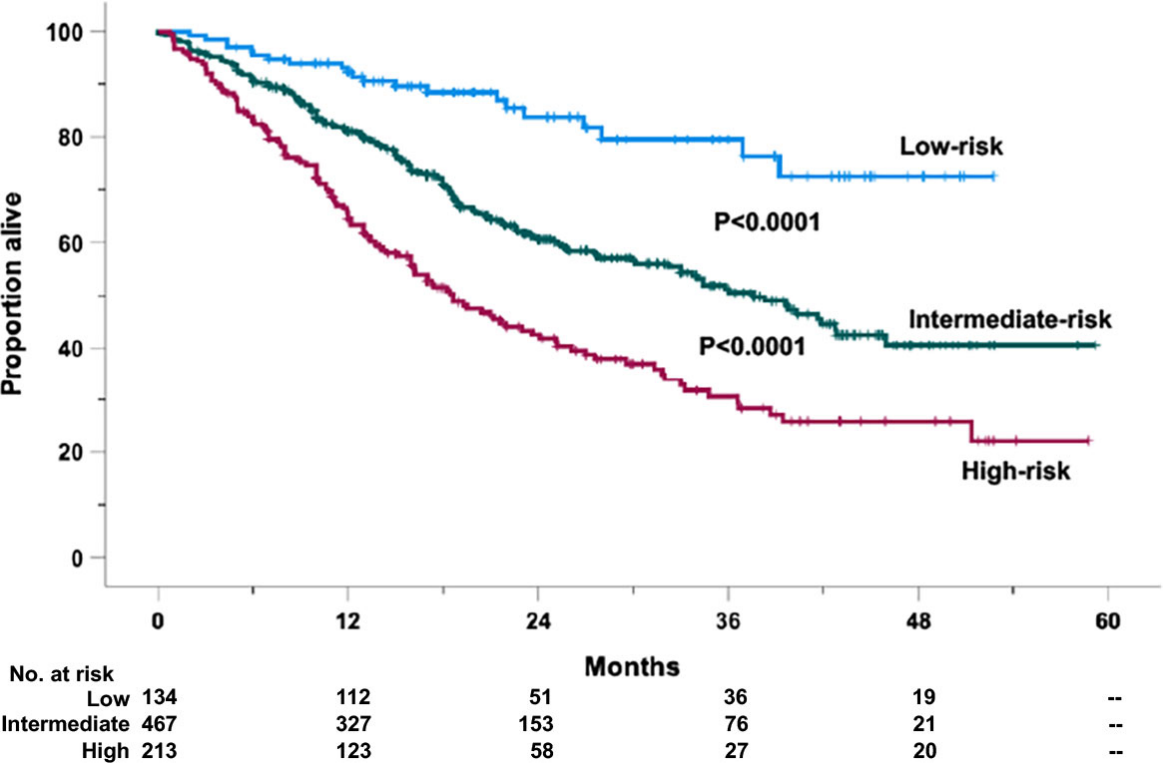
Overall survival (OS) of the retrospective relapsed/refractory (RR) multiple myeloma cases treated with KRd or EloRd clustered in low- (1st quartile, 134 cases), intermediate- (2nd–3rd quartiles, 467 cases), and high-risk categories (4th quartile, 213 cases), by the five-factor risk model. This analysis was carried out in 814 cases treated with KRd (559 cases) or EloRd (255 cases) in which all the five variables were available.

Utilizing the above detailed prognostic models, we derived a survival risk score for OS (SRS*^KRd/EloRd^*) and PFS (PRS*^KRd/EloRd^*). The regression coefficient in predicting mortality (29, 30) has been used to assign weights to variables independently associated with death or progression/death. The calculations of death and progression/death risk scores to be assigned on an individual basis are described in **Supplementary Tables 1**, **2**, respectively. These weights were summed up on an individual basis. Thus, a prognostic scale ranging from 0% (for patients unexposed to all risk factors) to 100% (for patients exposed to all risk factors) was identified in 814 cases. OS (**Supplementary Figure 6**) and PFS (**Supplementary Figure 7**) Kaplan–Meier curves were generated based on grouping cases by prognostic scale quartile. For OS, since the second quartile showed no significant difference with the third one, the two curves were gathered. Three risk categories were generated (**Figure 1**): low-risk (risk score < 25%), intermediate-risk (risk score ranging from 25% to 75%), and high-risk categories (risk score > 75%). Based on such stratification, 134 (16.5%) cases were allocated in the low-, 467 (57.3%) in the intermediate-, and 213 (26.2%) in the high-risk groups. The 1- and 2-year OS probability rates were 92.3% and 83.8% for the low-risk (HR = 1, reference category), 81.1% and 60.6% (HR = 2.73, 95% CI 1.73–4.28, *P* < 0.0001) for the intermediate-risk, and 65.5% and 42.5% (HR = 4.91, 95% CI 3.01–7.79, *P* < 0.0001) for the high-risk groups, respectively (**Figure 1**). Notably, different from the low-risk group, which did not cross the median timeline, the estimated median OS values were 36.6 ( ± 2.76 standard error of the mean) and 18.6 ( ± 1.93 standard error of the mean) months for the intermediate- and high-risk cases, respectively (**Figure 1**).

**Figure 2:**
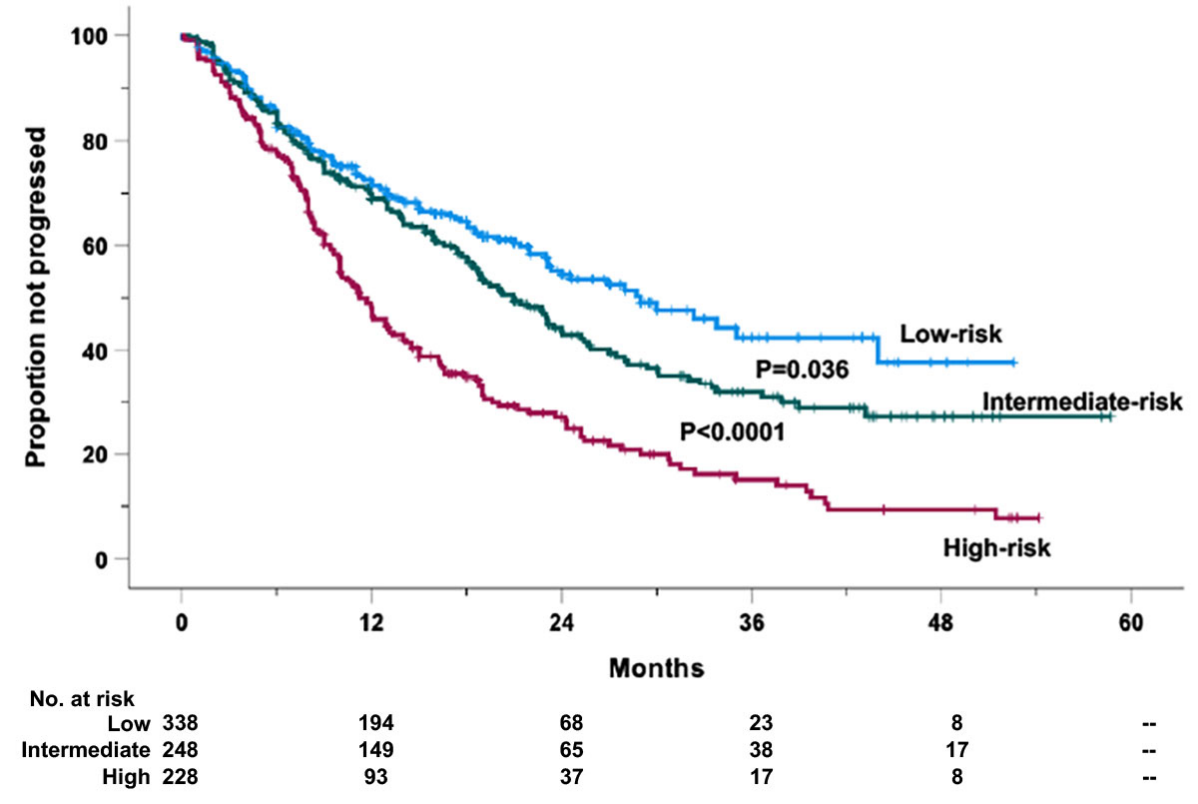
Progression-free survival (PFS) of the retrospective relapsed/refractory (RR) multiple myeloma cases treated with KRd or EloRd clustered in low- (1st and 2nd quartiles, 338 cases), intermediate- (2nd–3rd quartiles, 248 cases), high-risk categories (4th quartile, 228 cases), by the four-factor risk model. This analysis was carried out in 814 cases treated with KRd (559 cases) or EloRd (255 cases) in which all the five variables were available.

A similar procedure was followed for PFS. For PFS, since the second quartile showed no significant difference with the first one (**Supplementary Figure 7**), the two curves were clustered together. Again, three risk categories were generated (**Figure 2**): low-risk (risk score < 50%), intermediate-risk (risk score ranging from 50% to 75%), and high-risk categories (risk score > 75%). Based on such grouping, 338 (41.5%) cases were allocated in the low-, 248 (30.5%) in the intermediate-, and 228 (28.0%) in the high-risk groups. The 1- and 2-year PFS probability rates were 71.4% and 54.5% for the low-risk (HR = 1, reference category), 68.9% and 43.7% (HR = 1.95, 95% CI 1.40–2.72, *P* < 0.0001) for the intermediate-risk, and 48.0% and 27.1% (HR = 3.73, 95% CI 2.64–5.26, *P* < 0.0001) for the high-risk groups, respectively (**Figure 2**). The estimated median PFS values were 29.0 ( ± 3.70 standard error of the mean), 21.0 ( ± 1.52 standard error of the mean), and 11.7 ( ± 0.89 standard error of the mean) months for the low-, intermediate-, and high-risk cases, respectively (**Figure 2**).

Of note, the prognostic performance of the risk prediction rules for OS tended to be higher (*P* for trend = 0.05) according to the increase of previous lines of therapy. On the contrary, no difference in prognostic performance according to lines of therapy was found for the risk prediction rule for PFS (*P* for trend = 0.20). Of note, the two risk prediction rules had higher prognostic values for PFS (AUC: 66%) and OS (AUC: 65%) as compared to those of the other biomarkers/risk factors (PFS, AUC ranging from 54% to 62%; OS, AUC ranging from 53% to 61%) (**Table 2**).

**Table 2:** Median survival time (and 95% CI), hazard ratio (and 95% CI), and area under the ROC curve of the proposed risk prediction rules, the components from which they were built and the markers that were excluded.

Progression-free survival (PFS)	Median survival time (95% CI), months	Hazard ratio (95% CI)	Area under ROC curve

Cytogenetic risk	Normal: 27.5 (22.2–32.7) High: 8.4 (5.7–11.1)	2.60 (1.83–3.71), *P* < 0.001	0.58 ± 0.03 *P* = 0.014
PRS*^KRd/EloRd^*	Low risk: 29.0 (21.8–36.1) Intermediate risk: 21.0 (18.0–24.0) High risk: 11.7 (10.1–13.3)	1 (Ref.) 1.29 (1.01–1.63), *P* = 0.04 2.23 (1.78–2.80), *P* < 0.001	0.66 ± 0.02 *P* < 0.001
ISS	I–II: 22.7 (20.1–25.4) III: 12.9 (9.3–16.5)	1.47 (1.20–1.79), *P* < 0.001	0.55 ± 0.02 *P* = 0.02
Previous exposure to lenalidomide	No: 23.1 (20.2–25.9) Yes: 15.0 (12.1–17.9)	1.40 (1.17–1.67), *P* < 0.001	0.56 ± 0.02 *P* = 0.001
Age	<65.9 years: 28.7 (23.2–34.3) ≥65.9 years: 17.4 (15.2–19.6)	1.55 (1.29–1.87), *P* < 0.001	0.62 ± 0.02 *P* < 0.001
Number of previous lines of therapy	≤3: 23.0 (20.5–25.5) >3: 9.4 (7.3–11.6)	2.10 (1.66–2.67), *P* < 0.001	0.54 ± 0.02 *P* = 0.03
Overall survival (OS)			
Cytogenetic risk	Normal: 47.0 (NA–NA) High: 18.9 (10.5–27.3)	2.80 (1.83–4.30), *P* < 0.001	0.59 ± 0.04 *P* = 0.016
SRS*^KRd/EloRd^*	Low risk: not reached Intermediate risk: 37.6 (32.2–43.0) High risk: 18.6 (14.9–22.4)	1 (Ref.) 2.73 (1.74–4.28), *P* < 0.001 4.91 (3.09–7.79), *P* < 0.001	0.65 ± 0.02 *P* < 0.001
Interval diagnosis–therapy	≤3.5 years: 31.8 (26.8–36.9) >3.5 years: 39.8 (33.6–46.0)	0.77 (0.63–0.95), *P* = 0.015	0.53 ± 0.02 *P* = 0.10
ISS	I–II: 39.2 (33.9–44.6) III: 27.5 (20.1–34.9)	1.48 (1.18–1.86), *P* = 0.001	0.54 ± 0.02 *P* = 0.03
Previous exposure to lenalidomide	No: 38.3 (31.7–44.9) Yes: 33.0 (26.1–39.9)	1.25 (1.02–1.54), *P* = 0.036	0.54 ± 0.02 *P* = 0.04
Age	<65.5 years: not reached ≥65.5 years: 30.1 (25.9–34.3)	1.69 (1.3–2.1), *P* < 0.001	0.61 ± 0.02 *P* < 0.001
Disease status at KRd/EloRd start	Relapse: 37.6 (32.4–42.8) Refractory: 32.0 (25.3–38.7)	1.25 (1.01–1.55), *P* = 0.048	0.54 ± 0.02 *P* = 0.045

The concordance between the two scores is reported in **Supplementary Table 3**. The weighted kappa statistics between the risk classification provided by SRS*^KRd/EloRd^* and PRS*^KRd/EloRd^* was 48% (*P* < 0.001), indicating a moderate agreement between the two risk prediction rules.

Finally, a risk stratification derived by combining cases according to the risk categories of SRS and PRS (i.e., reference group, both SRS and PRS in the low-risk category; intermediate-risk group, SRS or PRS in the high-risk category; high-risk group, both SRS and PRS in the high-risk category) was performed. This analysis showed that patients with both SRS and PRS in the high-risk category had hazard ratios of progression/death and mortality that were 2.354 and 2.177 times higher, respectively, than those of the reference group and higher than those of the intermediate-risk group (**Supplementary Figures 8**, **9**).

### Impact of Cytogenetic Risk on Outcome

The FISH prognostic relevance, also emphasized by the R-ISS (12), encouraged us to perform an ancillary analysis in this small subgroup of patients. Univariate Cox analyses detected a significantly increased risk of death (HR = 2.80, 95% CI 1.82–4.30, *P* < 0.0001) and progression/death (HR = 2.60, 95% CI 1.83–3.71, *P* < 0.0001) for the cases that presented unfavorable cytogenetic abnormalities. When the five-factor SRS*^KRd/EloRd^* model and the four-factor PRS*^KRd/EloRd^* model were forced with FISH risk classification in two different multivariate analyses (**Supplementary Table 4**), cytogenetics maintained its independent negative prognostic role in predicting both death (HR = 2.68, 95% CI 1.72–4.20, *P* < 0.001) (**Supplementary Table 4a**) and progression/death (HR = 2.60, 95% CI 1.79–3.78, *P* < 0.001) (**Supplementary Table 4b**) likelihoods. Remarkably, also the five-factor SRS*^KRd/EloRd^* and the four-factor PRS*^KRd/EloRd^* scores were confirmed as independent predictors of OS and PFS in the statistical models including cytogenetic risk. Indeed, a dose–response increase of the hazard ratios of both mortality and progression/death was found in close parallelism with the rise of SRS*^KRd/EloRd^* and PRS*^KRd/EloRd^* (both *P* for trend < 0.001). The adjusted HRs of mortality in the intermediate- and high-risk categories were 2.48 (95% CI 0.89–6.86) and 5.62 (95% CI 2.0–15.8) times higher, respectively, than those in the low-risk category (reference group), and this was also true for progression/death (intermediate versus reference category, hazard ratio 1.17, 95% CI 0.78–1.76; high versus reference category, hazard ratio 2.42, 95% CI 1.63–3.59) (**Supplementary Tables 4a,b**). These results indicate that cytogenetic risk and the two risk scores (SRS*^KRd/EloRd^* and PRS*^KRd/EloRd^*) have a complementary role to predict OS and PFS in the study population.

## Discussion

New-generation PIs, IMIs, with or without conventional chemotherapy, are covering the treatment landscape of RRMM (11). However, despite therapeutic algorithms, MM patients ultimately relapse. Risk stratification in a newly diagnosed MM, relying on preinduction characteristics, remains a crucial model both for patient counseling and the risk-adapted therapeutic strategy progress (33).

Nevertheless, few studies have been conducted with the aim of identifying prognostic factors for predicting clinical outcomes following specific treatments in the setting of RRMM patients. Recently, in a Chinese MM cohort involving RRMM patients treated with anti-BCMA CAR-T cell therapy, a Cox model based on extramedullary disease, light-chain MM, high-risk cytogenetics, and more than three therapeutic lines allowed the early identification of cases with poor PFS (34). A powerful emerging predictor is the minimal residual disease assessment (35). However, the risk stratification for RRMM patients treated with new combinations is missing and desirable to guide sequential therapy choices better.

In this retrospective real-world study, we exploit a scoring system to foresee outcomes in MM patients receiving the two most common triplets, KRd (16, 17) or EloRd (18, 19), as salvage therapy outside of clinical trials. Using readily accessible clinical data at the time of the two triplets’ start, these scores identified low-, intermediate-, and high-risk groups. The analyses showed 2.7- and 4.9-fold increased risk of death for the intermediate- and high-risk groups, respectively. Moreover, the progression risks after salvage therapy with the two triplets were 1.3 and 2.2 times higher for cases clustered in the intermediate- and high-risk groups, respectively.

Although the most common tools used for risk stratification in MM are the ISS (31) and, more recently, the R-ISS (12), none were explicitly generated for real-life cohorts of patients treated with salvage therapy with KRd/EloRd. In this respect, it was reasonable to consider that in addition to ISS, a more stringent focus on specific patient subgroups, such as those exposed to lenalidomide, would help improve prognosis.

Accordingly, age, previous lines of therapies, and prior lenalidomide exposure with the well-known ISS had represented the backbone of both SRS*^KRd/EloRd^* and PRS*^KRd/EloRd^* models. Moreover, refractoriness status at KRd/EloRd start was an additional variable in the prognostication clustering for OS.

In our analysis, older age was a critical concern since it surrogates comorbidities and worse fitness status (36). However, the use of age only could be reductive, and an adequate assessment of fitness status before treatment remains remarkably critical before planning salvage therapy in MM. Although the current physician’s effort is to reduce the risk of either over- or undertreating respectively unfit and fit patients, the routine application of frailty scores is objectively less applicable in some real-world hardworking clinical settings (37).

The RRMM treatment that had three or more previous lines of therapy, or exposed/refractory to lenalidomide, or refractory to prior therapy before the salvage, is becoming exceptionally challenging (11). In this respect, regimens containing daratumumab (38), isatuximab (39–41), or elotuzumab (42) could represent reasonable therapeutic options. Nevertheless, due to its exclusive many-sided mechanisms of action, daratumumab is increasingly used in treating RRMM and newly diagnosed cases. Alongside its efficacy and low-toxicity profile, daratumumab manageability is enhanced further with the subcutaneous formulation (43). However, the increasing use of daratumumab in the first line will shortly develop a new task in RRMM relapsing patients while on anti-CD38 treatment (44).

Unlike the ISS model, which uses two routine and inexpensive laboratory pieces (31), the R-ISS added cytogenetic analysis and LDH levels (12). This latter variable revealed a borderline significance in univariate analysis and was thus excluded from the final model. A substantial number of cytogenetic studies were lacking in this cohort, mirroring the scarce attitude in performing this assay in the real-world setting. The available cases were only 32.5%, with less than one-quarter presenting unfavorable cytogenetic abnormalities. Although with this limitation, we performed an ancillary analysis yet. Interestingly, SRS*^KRd/EloRd^* and PRS*^KRd/EloRd^* scores were confirmed as independent predictors of OS and PFS in the statistical models, including cytogenetic risk, indicating that cytogenetics and both our risk scores might have a complementary prognostic role in envisaging overall and progression-free survival in the study cohort. A limitation of our findings is the lack of external validation. Thus, our results need to be confirmed in an independent cohort of RRMM patients.

Furthermore, the validation of our new risk prediction score requires a clinical validation, i.e., a randomized trial testing whether a treatment strategy guided by our risk prediction rule preludes to a better prognosis as compared to a treatment strategy based on previous prognostic tools or standard of care.

Currently, in clinical practice, patients who relapse after KRd and EloRd receive a therapeutic regimen based on pomalidomide (isatuximab, pomalidomide, and dexamethasone or elotuzumab, pomalidomide, and dexamethasone) (38, 42), these approaches represent a promising therapeutic strategy for the standard risk category. If the results of this study will be confirmed in an external validation cohort, patients at high risk for both scores should be candidates for innovative therapies.

Remarkably, a feature triple-class-exposed or even refractory RRMM cohort is realistically expected, especially in developed countries. Thus, new standards of care, i.e., drugs targeting B-cell maturation antigen, chimeric antigen receptor T cells, antibody–drug conjugate, bispecific T-cell engager, and a bispecific antibody (45), will be hopefully incorporated into the RRMM treatment algorithm to dodge the next drug resistances.

At this point, the clinical and laboratory criteria utilized for building the score systems above cannot comprehensively capture the molecular and biological indicators underlying the resistance machinery of new treatments and, ultimately, the PFS and OS magnitude. In this respect, the use of different analytical methods to establish a comprehensive prognostic scoring system, including gene expression/mutation-derived risk scores and clinical prognostic signatures, is desirable to improve predictive precision and guide future clinical therapy (46, 47). Nevertheless, next-generation markers are far from their systematic utilization in RRMM real-world patients.

## Data Availability Statement

The raw data supporting the conclusions of this article will be made available by the authors, without undue reservation.

## Ethics Statement

This study was reviewed and approved by Ethic Committee of Cosenza, 2021/106. The patients/participants provided their written informed consent to participate in this study.

## Author Contributions

FMo, EZ, AN, and MGe designed the study. FMo and GT performed the statistical analysis. FMo, GT, AN, and MGe analyzed and interpreted the data. FMe, EZ, GT, AN, and MGe wrote the manuscript. CCo, VP, SP, SBr, MGa, SM, SMi, DD, ER, RR, LC, PT, GM, IV, EM, AB, FMe, EV, CB, AM, LP, SR, BGar, NC, SBa, FF, PC, AF, CCa, GR, GF, MBa, IR, PM, VS, MM, MP, MO, FR, MBo, MC, and MGe provided the patients and collected the clinical data. All authors gave final approval for the manuscript.

## Funding

The study was partially funded by the Italian Ministry of Health—Ricerca Corrente 2023 (to AN).

## Conflict of Interest

PT received honoraria from Bristol-Myers Squibb, Takeda, Janssen, Celgene, Amgen; LP received honoraria from Celgene, Takeda, Janssen, Amgen; EZ has received a speaker honorarium from Bristol-Myers Squibb, Takeda, Janssen, Celgene, Amgen; MC has received a speaker honorarium from Adaptive Biotechnology, Amgen, Bristol-Myers Squibb, Celgene, Janssen, Takeda, AbbVie, GlaxoSmithKline and he is a member of committee of these Company; CC and FDR received honoraria from Amgen. CC, and FDR received honoraria from Celgene.

The remaining authors declare that the research was conducted in the absence of any commercial or financial relationships that could be construed as a potential conflict of interest.

## Publisher’s Note

All claims expressed in this article are solely those of the authors and do not necessarily represent those of their affiliated organizations, or those of the publisher, the editors and the reviewers. Any product that may be evaluated in this article, or claim that may be made by its manufacturer, is not guaranteed or endorsed by the publisher.

## References

[1] Kumar SK Dispenzieri A Lacy MQ Gertz MA Buadi FK Pandey S. Continued Improvement in Survival in Multiple Myeloma: Changes in Early Mortality and Outcomes in Older Patients. Leukemia (2014) 28(5):1122–8. doi: 10.1038/leu.2013.313 PMC4000285 24157580

[2] Kumar SK Lee JH Lahuerta JJ Morgan G Richardson PG Crowley J. Risk of Progression and Survival in Multiple Myeloma Relapsing After Therapy With IMiDs and Bortezomib: A Multicenter International Myeloma Working Group Study. Leukemia (2012) 26(1):149–57. doi: 10.1038/leu.2011.196 PMC4109061 21799510

[3] Kumar SK Dimopoulos MA Kastritis E Terpos E Nahi H Goldschmidt H. Natural History of Relapsed Myeloma, Refractory to Immunomodulatory Drugs and Proteasome Inhibitors: A Multicenter IMWG Study. Leukemia (2017) 31(11):2443–8. doi: 10.1038/leu.2017.138 28620163

[4] Usmani S Ahmadi T Ng Y Lam A Desai A Potluri R. Analysis of Real-World Data on Overall Survival in Multiple Myeloma Patients With ≥3 Prior Lines of Therapy Including a Proteasome Inhibitor (PI) and an Immunomodulatory Drug (IMiD), or Double Refractory to a PI and an IMiD. Oncologist (2016) 21(11):1355–61. doi: 10.1634/theoncologist.2016-0104 PMC5189616 27486203

[5] Yong K Delforge M Driessen C Fink L Flinois A Gonzalez-McQuire S. Multiple Myeloma: Patient Outcomes in Real-World Practice. Br J Haematol (2016) 175(2):252–64. doi: 10.1111/bjh.14213 PMC5096152 27411022

[6] Kumar SK Therneau TM Gertz MA Lacy MQ Dispenzieri A Rajkumar SV. Clinical Course of Patients With Relapsed Multiple Myeloma. Mayo Clin Proc (2004) 79(7):867–74. doi: 10.4065/79.7.867 15244382

[7] Legarda MA Cejalvo MJ de la Rubia J. Recent Advances in the Treatment of Patients With Multiple Myeloma. Cancers (Basel) (2020) 12(12):3576. doi: 10.3390/cancers12123576 PMC7761116 33265952

[8] Rajkumar SV Kumar S. Multiple Myeloma Current Treatment Algorithms. Blood Cancer J (2020) 10(9):94. doi: 10.1038/s41408-020-00359-2 32989217 PMC7523011

[9] Lee JH Kim SH. Treatment of Relapsed and Refractory Multiple Myeloma. Blood Res (2020) 55(S1):S43–53. doi: 10.5045/br.2020.S008 PMC7386890 32719176

[10] Durer C Durer S Lee S Chakraborty R Malik MN Rafae A. Treatment of Relapsed Multiple Myeloma: Evidence-Based Recommendations. Blood Rev (2020) 39:100616. doi: 10.1016/j.blre.2019.100616 31500848

[11] Moreau P Kumar SK San Miguel J Davies F Zamagni E Bahlis N. Treatment of Relapsed and Refractory Multiple Myeloma: Recommendations From the International Myeloma Working Group. Lancet Oncol (2021) 22(3):e105–18. doi: 10.1016/S1470-2045(20)30756-7 33662288

[12] Palumbo A Avet-Loiseau H Oliva S Lokhorst HM Goldschmidt H Rosinol L. Revised International Staging System for Multiple Myeloma: A Report From International Myeloma Working Group. J Clin Oncol (2015) 33(26):2863–9. doi: 10.1200/JCO.2015.61.2267 PMC4846284 26240224

[13] Sun Z Zheng F Wu S Liu Y Guo H Liu Y. Triplet Versus Doublet Combination Regimens for the Treatment of Relapsed or Refractory Multiple Myeloma: A Meta-Analysis of Phase III Randomized Controlled Trials. Crit Rev Oncol Hematol (2017) 113:249–55. doi: 10.1016/j.critrevonc.2017.03.018 28427514

[14] van Beurden-Tan CHY Franken MG Blommestein HM Uyl-de Groot CA Sonneveld P. Systematic Literature Review and Network Meta-Analysis of Treatment Outcomes in Relapsed and/or Refractory Multiple Myeloma. J Clin Oncol (2017) 35(12):1312–9. doi: 10.1200/JCO.2016.71.1663 28240968

[15] Quach H Ritchie D Stewart AK Neeson P Harrison S Smyth MJ. Mechanism of Action of Immunomodulatory Drugs (IMiDS) in Multiple Myeloma. Leukemia (2010) 24(1):22–32. doi: 10.1038/leu.2009.236 19907437 PMC3922408

[16] Stewart AK Rajkumar SV Dimopoulos MA Masszi T Špička I Oriol A. Carfilzomib, Lenalidomide, and Dexamethasone for Relapsed Multiple Myeloma. N Engl J Med (2015) 372(2):142–52. doi: 10.1056/NEJMoa1411321 25482145

[17] Siegel DS Dimopoulos MA Ludwig H Facon T Goldschmidt H Jakubowiak A. Improvement in Overall Survival With Carfilzomib, Lenalidomide, and Dexamethasone in Patients With Relapsed or Refractory Multiple Myeloma. J Clin Oncol (2018) 36(8):728–34. doi: 10.1200/JCO.2017.76.5032 29341834

[18] Lonial S Dimopoulos M Palumbo A White D Grosicki S Spicka I. Elotuzumab Therapy for Relapsed or Refractory Multiple Myeloma. N Engl J Med (2015) 373(7):621–31. doi: 10.1056/NEJMoa1505654 26035255

[19] Dimopoulos MA Lonial S Betts KA Chen C Zichlin ML Brun A. Elotuzumab Plus Lenalidomide and Dexamethasone in Relapsed/Refractory Multiple Myeloma: Extended 4-Year Follow-Up and Analysis of Relative Progression-Free Survival From the Randomized ELOQUENT-2 Trial. Cancer (2018) 124(20):4032–43. doi: 10.1002/cncr.31680 30204239

[20] Dimopoulos MA Oriol A Nahi H San-Miguel J Bahlis NJ Usmani SZ. Daratumumab, Lenalidomide, and Dexamethasone for Multiple Myeloma. N Engl J Med (2016) 375(14):1319–31. doi: 10.1056/NEJMoa1607751 27705267

[21] Dimopoulos MA San-Miguel J Belch A White D Benboubker L Cook G. Daratumumab Plus Lenalidomide and Dexamethasone Versus Lenalidomide and Dexamethasone in Relapsed or Refractory Multiple Myeloma: Updated Analysis of POLLUX. Haematologica (2018) 103(12):2088–96. doi: 10.3324/haematol.2018.194282 PMC6269302 30237262

[22] Conticello C Romano A Del Fabro V Martino EA Calafiore V Sapienza G. Et Al, Tolerability and Efficacy of Carfilzomib in Combination With Lenalidomide and Dexamethasone in Relapsed Refractory Myeloma Patients: A Retrospective Real-Life Survey of the Sicilian Myeloma Network. J Clin Med (2019) 8(6):877. doi: 10.3390/jcm8060877 PMC6617295 31248142

[23] Mele A Prete E De Risi C Citiso S Greco G Falcone AP. Carfilzomib, Lenalidomide, and Dexamethasone in Relapsed/Refractory Multiple Myeloma Patients: The Real-Life Experience of Rete Ematologica Pugliese (REP). Ann Hematol (2021) 100(2):429–36. doi: 10.1007/s00277-020-04329-3 33161453

[24] Rocchi S Tacchetti P Pantani L Mancuso K Rizzello I di Giovanni Bezzi C. A Real-World Efficacy and Safety Analysis of Combined Carfilzomib, Lenalidomide, and Dexamethasone (KRd) in Relapsed/Refractory Multiple Myeloma. Hematol Oncol (2021) 39(1):41–50. doi: 10.1002/hon.2820 33085797

[25] Palmieri S Rocco S Vitagliano O Catalano L Cerchione C Vincelli ID. KRD (Carfilzomib and Lenalidomide Plus Dexamethasone) for the Treatment of Relapsed or Refractory Multiple Myeloma in the Real-Life: A Retrospective Survey in 123 Patients. Ann Hematol (2020) 99(12):2903–9. doi: 10.1007/s00277-020-04158-4 32583088

[26] Martino EA Conticello C Zamagni E Pavone V Palmieri S Musso M. Carfilzomib Combined With Lenalidomide and Dexamethasone (KRd) as Salvage Therapy for Multiple Myeloma Patients: Italian, Multicenter, Retrospective Clinical Experience With 600 Cases Outside of Controlled Clinical Trials. Hematol Oncol (2022). doi: 10.1002/hon.3035 35638723

[27] Gentile M Specchia G Derudas D Galli M Botta C Rocco S. Elotuzumab, Lenalidomide, and Dexamethasone as Salvage Therapy for Patients With Multiple Myeloma: Italian, Multicenter, Retrospective Clinical Experience With 300 Cases Outside of Controlled Clinical Trials. Haematologica (2021) 106(1):291–4. doi: 10.3324/haematol.2019.241513 PMC7776255 32107338

[28] Bruzzese A Derudas D Galli M Martino EA Rocco S Conticello C. Elotuzumab Plus Lenalidomide and Dexamethasone in Relapsed/Refractory Multiple Myeloma: Extended 3-Year Follow-Up of a Multicenter, Retrospective Clinical Experience With 319 Cases Outside of Controlled Clinical Trials. Hematol Oncol (2022). doi: 10.1002/hon.3031 35608183

[29] Morabito F Zamagni E Conticello C Pavone V Palmieri S Bringhen S. Adjusted Comparison Between Elotuzumab and Carfilzomib in Combination With Lenalidomide and Dexamethasone as Salvage Therapy for Multiple Myeloma Patients. Eur J Haematol (2021) 108(3):178–89. doi: 10.1111/ejh.13723 34716957

[30] Kumar S Paiva B Anderson KC Durie B Landgren O Moreau P. International Myeloma Working Group Consensus Criteria for Response and Minimal Residual Disease Assessment in Multiple Myeloma. Lancet Oncol (2016) 17:e328–46. doi: 10.1016/S1470-2045(16)30206-6 27511158

[31] Greipp PR San Miguel J Durie BG Crowley JJ Barlogie B Bladé J. International Staging System for Multiple Myeloma. J Clin Oncol (2005) 23(15):3412–20. doi: 10.1200/JCO.2005.04.242 15809451

[32] Morabito F Tripepi G Moia R Recchia AG Boggione P Mauro FR. Lymphocyte Doubling Time As A Key Prognostic Factor To Predict Time To First Treatment In Early-Stage Chronic Lymphocytic Leukemia. Front Oncol (2021) 11:684621. doi: 10.3389/fonc.2021.684621 34408978 PMC8366564

[33] Chng WJ Dispenzieri A Chim CS Fonseca R Goldschmidt H Lentzsch S. IMWG Consensus on Risk Stratification in Multiple Myeloma. Leukemia (2014) 28:269–77. doi: 10.1038/leu.2013.247 23974982

[34] Munshi NC Avet-Loiseau H Anderson KC Neri P Paiva B Samur M. A Large Meta-Analysis Establishes the Role of MRD Negativity in Long-Term Survival Outcomes in Patients With Multiple Myeloma. Blood Adv (2020) 4:5988–99. doi: 10.1182/bloodadvances.2020002827 PMC7724898 33284948

[35] Zweegman S Engelhardt M Larocca A. EHA SWG on ‘Aging and Hematology’. Elderly Patients With Multiple Myeloma: Towards a Frailty Approach? Curr Opin Oncol (2017) 29(5):315–21. doi: 10.1097/CCO.0000000000000395 28763310

[36] Möller MD Gengenbach L Graziani G Greil C Wäsch R Engelhardt M. Geriatric Assessments and Frailty Scores in Multiple Myeloma Patients: A Needed Tool for Individualized Treatment? Curr Opin Oncol (2021) 33(6):648–57. doi: 10.1097/CCO.0000000000000792 PMC8528138 34534141

[37] Dimopoulos MA Terpos E Boccadoro M Delimpasi S Beksac M Katodritou E. APOLLO Trial Investigators. Daratumumab Plus Pomalidomide and Dexamethasone Versus Pomalidomide and Dexamethasone Alone in Previously Treated Multiple Myeloma (APOLLO): An Open-Label, Randomised, Phase 3 Trial. Lancet Oncol (2021) 22(6):801–12. doi: 10.1016/S1470-2045(21)00128-5 34087126

[38] Frampton JE Attal M Richardson PG Rajkumar SV San-Miguel J Beksac M. Isatuximab Plus Pomalidomide and Low-Dose Dexamethasone Versus Pomalidomide and Low-Dose Dexamethasone in Patients With Relapsed and Refractory Multiple Myeloma (ICARIA-MM): A Randomised, Multicentre, Open-Label, Phase 3 Study. Lancet (2019) 394(10214):2096–107. doi: 10.1016/S0140-6736(19)32556-5 31735560

[39] Moreau P Dimopoulos MA Mikhael J Yong K Capra M Facon T. Isatuximab, Carfilzomib, and Dexamethasone in Relapsed Multiple Myeloma (IKEMA): A Multicentre, Open-Label, Randomised Phase 3 Trial. Lancet (2021) 397(10292):2361–71. doi: 10.1016/S0140-6736(21)00592-4 34097854

[40] Bringhen S Pour L Vorobyev V Vural F Warzocha K Benboubker L. Isatuximab Plus Pomalidomide and Dexamethasone in Patients With Relapsed/Refractory Multiple Myeloma According to Prior Lines of Treatment and Refractory Status: ICARIA-MM Subgroup Analysis. Leuk Res (2021) 104:106576. doi: 10.1016/j.leukres.2021.106576 33839618

[41] Mateos MV Nahi H Legiec W Grosicki S Vorobyev V Spicka I. Subcutaneous Versus Intravenous Daratumumab in Patients With Relapsed or Refractory Multiple Myeloma (COLUMBA): A Multicentre, Open-Label, Non-Inferiority, Randomised, Phase 3 Trial. Lancet Haematol (2020) 7(5):e370–80. doi: 10.1016/S2352-3026(20)30070-3 32213342

[42] Dimopoulos MA Dytfeld D Grosicki S Moreau P Takezako N Hori M. Elotuzumab Plus Pomalidomide and Dexamethasone for Multiple Myeloma. N Engl J Med (2018) 379(19):1811–22. doi: 10.1056/NEJMoa1805762 30403938

[43] Kastritis E Terpos E Dimopoulos MA. How I Treat Relapsed Multiple Myeloma. Blood (2022) 139(19):2904–17. doi: 10.1182/blood.2020008734 35007326

[44] Mosquera Orgueira A González Pérez MS Díaz Arias JÁ Antelo Rodríguez B Alonso Vence N Bendaña López Á. Survival Prediction and Treatment Optimization of Multiple Myeloma Patients Using Machine-Learning Models Based on Clinical and Gene Expression Data. Leukemia (2021) 35(10):2924–35. doi: 10.1038/s41375-021-01286-2 34007046

[45] Cho SF Xing L Anderson KC Tai YT. Promising Antigens for the New Frontier of Targeted Immunotherapy in Multiple Myeloma. Cancers (Basel) (2021) 13(23):6136. doi: 10.3390/cancers13236136 34885245 PMC8657018

[46] Soliman AM Das S Teoh SL. Next-Generation Biomarkers in Multiple Myeloma: Understanding the Molecular Basis for Potential Use in Diagnosis and Prognosis. Int J Mol Sci (2021) 22(14):7470. doi: 10.3390/ijms22147470 34299097 PMC8305153

[47] Liu L Qu J Dai Y Qi T Teng X Li G. An Interactive Nomogram Based on Clinical and Molecular Signatures to Predict Prognosis in Multiple Myeloma Patients. Aging (Albany NY) (2021) 13(14):18442–63. doi: 10.18632/aging.203294 PMC8351694 34260414

