# Supplementary material for: Survival Risk Scores for Real-Life Relapsed/Refractory Multiple Myeloma Patients Receiving Elotuzumab or Carfilzomib In Combination With Lenalidomide and Dexamethasone as Salvage Therapy: Analysis of 919 Cases Outside Clinical Trials
Source: Front Oncol. 2022 Jul 18;12:890376. doi: 10.3389/fonc.2022.890376 (PMC9341470; doi:10.3389/fonc.2022.890376)
Supplement: Supplementary file 1 [file DataSheet_1.docx]

Supplementary Material

# Supplementary Tables

| **Table S1.** Regression coefficients (b), percentage weights (%), and the overall survival risk score calculation derived from the 5-factor multivariate model. This analysis was carried out in 814 relapsed/refractory multiple myeloma (RRMM) cases treated with KRd (n=559 cases) or EloRd (n=255), in which all the variables were available. | | | |
| --- | --- | --- | --- |
| **Variables** | **Regression coefficients (b)** | **Score**  **calculation** | **Risk**  **scores (%)** |
| **Interval diagnosis-therapy, >3.5 years** | 0.383 | 0.383/2.154 = 0.178 | < 3.5 = 0  > 3.5 = 17.8 |
| **Prior exposure to lenalidomide** | 0.264 | 0.264/2.154 = 0.123 | No = 0  Yes = 12.3 |
| **International Stage System (ISS), III** | 0.274 | 0.274/2.154 = 0.127 | I-II = 0  III = 12.7 |
| **Age, > 65.5 years** | 0.547 | 0.547/2.154 = 0.254 | < 65.5 = 0  > 65.5 = 25.4 |
| **Number of previous lines of therapies** | 0.686 | 0.686/2.154 = 0.318 | < 3 = 0  > 3 = 31.8 |
|  | ***Total=2.154** |  |  |

| **Table S2.** Regression coefficients (b), percentage weights (%), and the progression-free survival risk score calculation derived from the 4-factor multivariate model. This analysis was carried out in 814 relapsed/refractory multiple myeloma (RRMM) cases treated with KRd (n=559 cases) or EloRd (n=255), in which all the variables were available. | | | |
| --- | --- | --- | --- |
| **Variables** | **Regression coefficients (b)** | **Score**  **calculation** | **Risk**  **scores (%)** |
| **Prior exposure to lenalidomide** | 0.298 | 0.298/1.614 = 0.185 | No = 0  Yes = 18.5 |
| **International Stage System (ISS), III** | 0.312 | 0.312/1.614 = 0.193 | I-II = 0  III = 19.3 |
| **Age, > 65.9 years** | 0.493 | 0.493/1.614 = 0.305 | < 65.9 = 0  > 65.9 = 30.5 |
| **Number of previous lines of therapies** | 0.511 | 0.511/1.614 = 0.317 | < 3 = 0  > 3 = 31.7 |
|  | ***Total=1.614** |  |  |

| \| **Table S3.** Relationship between overall (SRS*^KRd/EloRd^*) and progression-free (PRS*^KRd/EloRd^*) survival prognostic scores. \| \| \| \| \| \| \| --- \| --- \| --- \| --- \| --- \| --- \| \|  \|  \| PRS*^KRd/EloRd^* \|  \|  \|  \| \|  \|  \| low-risk  N° of cases (%) \| intermediate-risk  N° of cases (%) \| high-risk  N° of cases (%) \| Total \| \| SRS*^KRd/EloRd^* \| low-risk  N° of cases (%) \| 134 (100) \| --- \| --- \| 134 \| \|  \| intermediate-risk N° of cases (%) \| 180 (38.5) \| 226 (48.4) \| 61 (13.1) \| 467 \| \|  \| high-risk  N° of cases (%) \| 24 (11.3) \| 22 (10.3) \| 167 (78.4) \| 213 \|   **Table S4.** Cox regression multivariate analyses in which survival risk scores for all-cause mortality (a) and disease progression or death (b) were adjusted for cytogenetic risk. This analysis was carried out in 279 relapsed/refractory multiple myeloma (RRMM) cases treated with KRd (n=225 cases) or EloRd (n=54), in which all the variables were available | | | |
| --- | --- | --- | --- | --- | --- | --- | --- | --- | --- | --- | --- | --- | --- | --- | --- | --- | --- | --- | --- | --- | --- | --- | --- | --- | --- | --- | --- | --- | --- | --- | --- | --- | --- | --- | --- | --- | --- | --- | --- |
| **a)** | **All-cause mortality** | | |
| **Covariates** | **Hazard Ratio** | **95% Confidential Interval** | **P for trend** |
| Risk categories |  |  |  |
| 1 | 1* | … | <0.001 |
| 2 | 2.48 | 0.89-6.86 |  |
| 3 | 5.62 | 2.00-15.76 |  |
| Cytogenetic risk | 2.68 | 1.72-4.20 | <0.001 |

| **b)** | **Disease Progression or Death** | | |
| --- | --- | --- | --- |
| **Covariates** | **Hazard Ratio** | **95% Confidential Interval** | **P for trend** |
| Risk categories |  |  |  |
| 1 | 1* | … | <0.001 |
| 2 | 1.17 | 0.78-1.76 |  |
| 3 | 2.42 | 1.63-3.59 |  |
| Cytogenetic risk | 2.60 | 1.79-3.77 | <0.001 |

*Reference group.


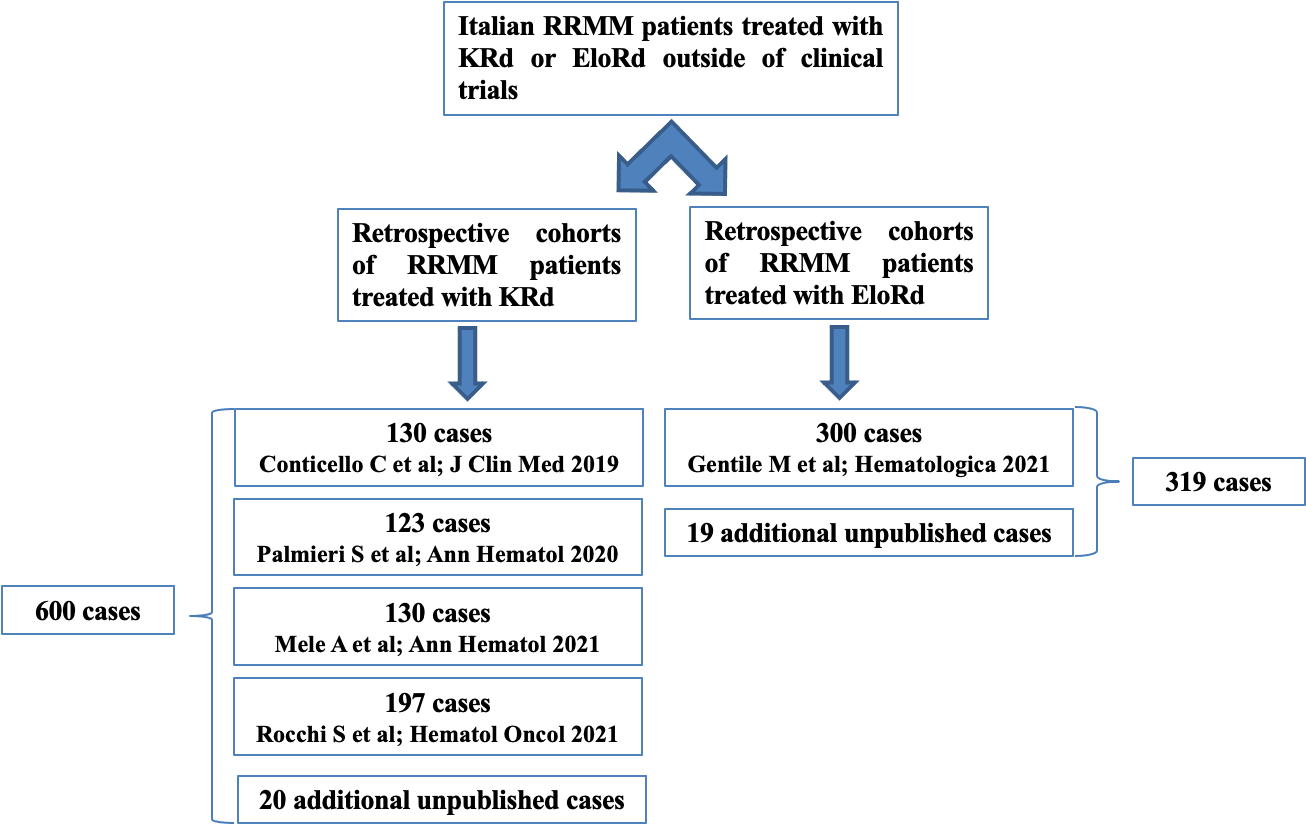


**Supplementary Figure 1.** Flow Diagram of the progress through the enrollment phases of the five real-world cohorts.


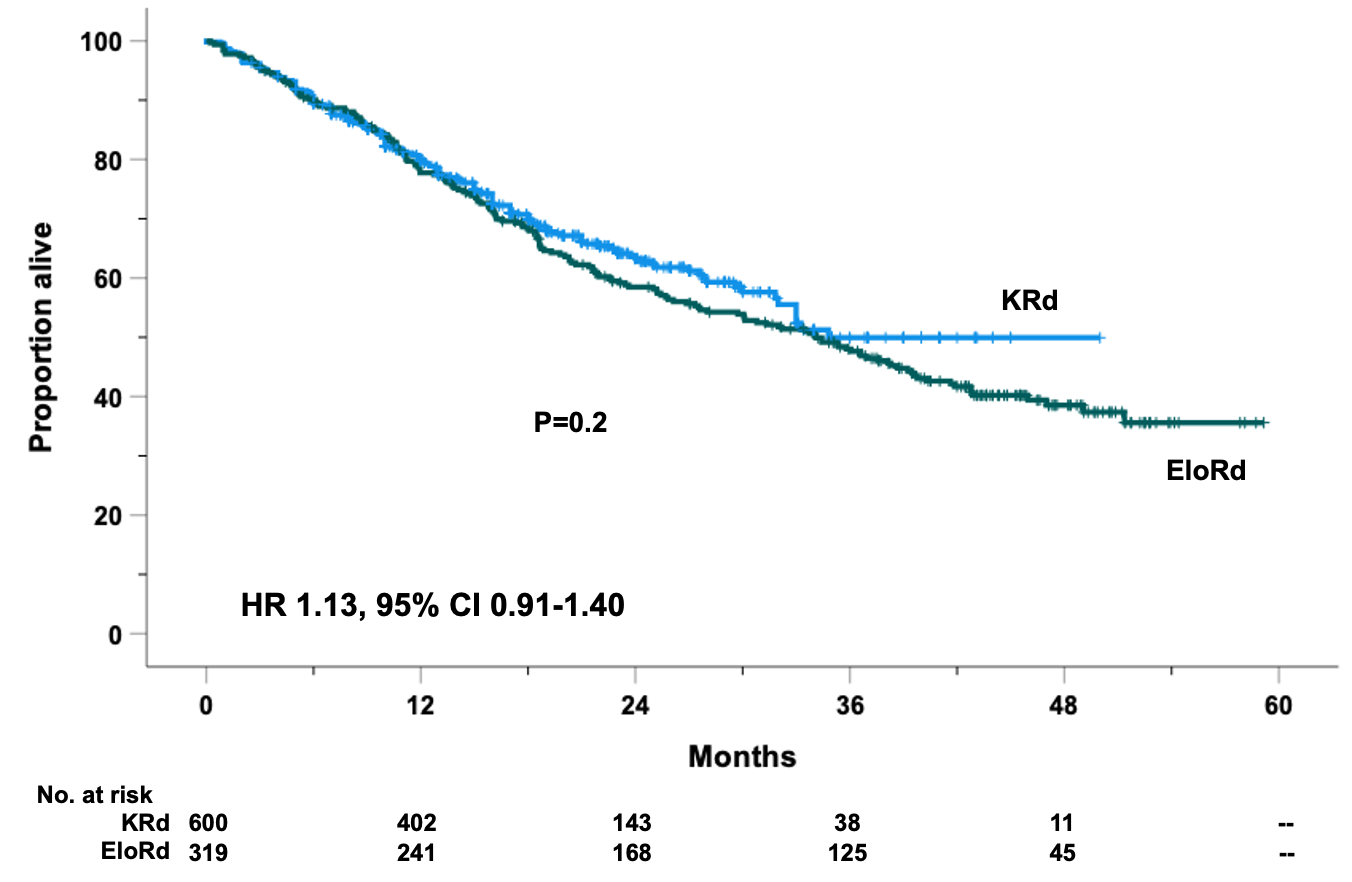


**Supplementary Figure 2.** Overall survival (OS) of the retrospective relapsed/refractory (RR) multiple myeloma cases clustered by KRd [(Carlfizomib (K), lenalidomide (R), dexamethasone (d)] *versus* EloRd [Elotuzumab (Elo) Rd].

**
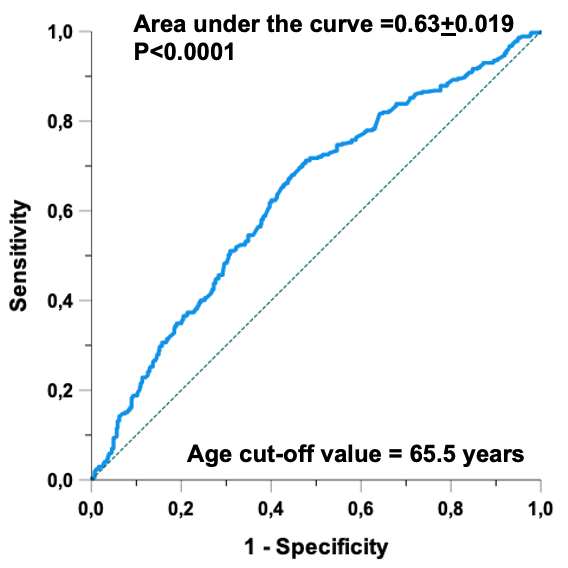
**

**Supplementary Figure 3.** Receiver Operating Characteristic (ROC) analysis of age to identify patients who died. The dashed line represents the reference line of prognostic usefulness.


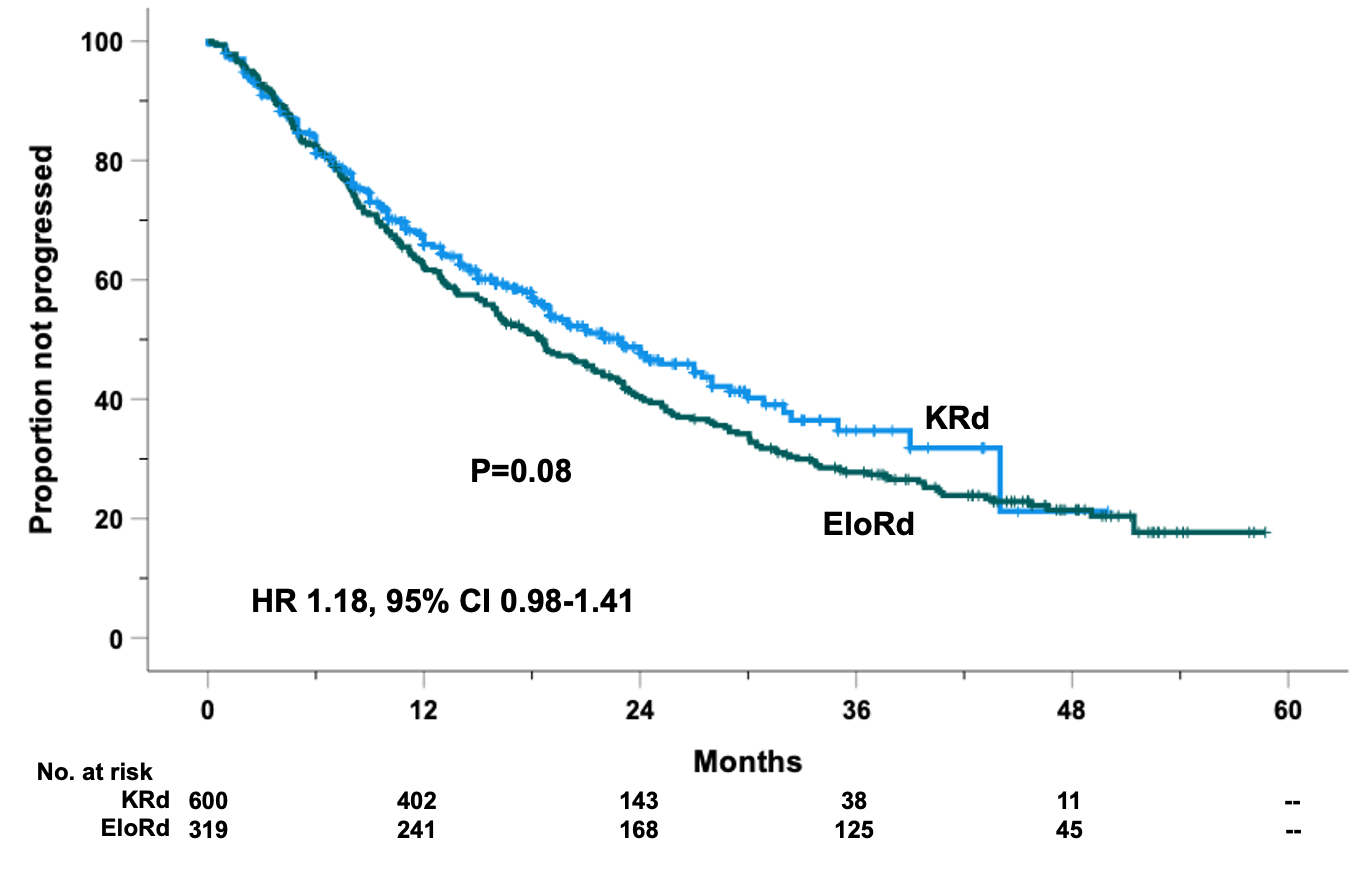


**Supplementary Figure 4.** Progression-free survival (PFS) of the retrospective relapsed/refractory (RR) multiple myeloma cases clustered by KRd [(Carlfizomib (K), lenalidomide (R), dexamethasone (d)] versus EloRd [Elotuzumab (Elo) Rd].

**
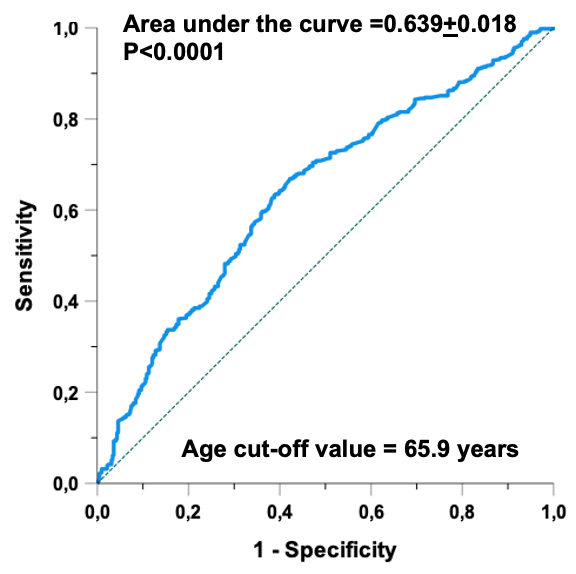
**

**Supplementary Figure 5.** Receiver Operating Characteristic (ROC) analysis of age to identify patients who progressed or died. The dashed line represents the reference line of prognostic usefulness.


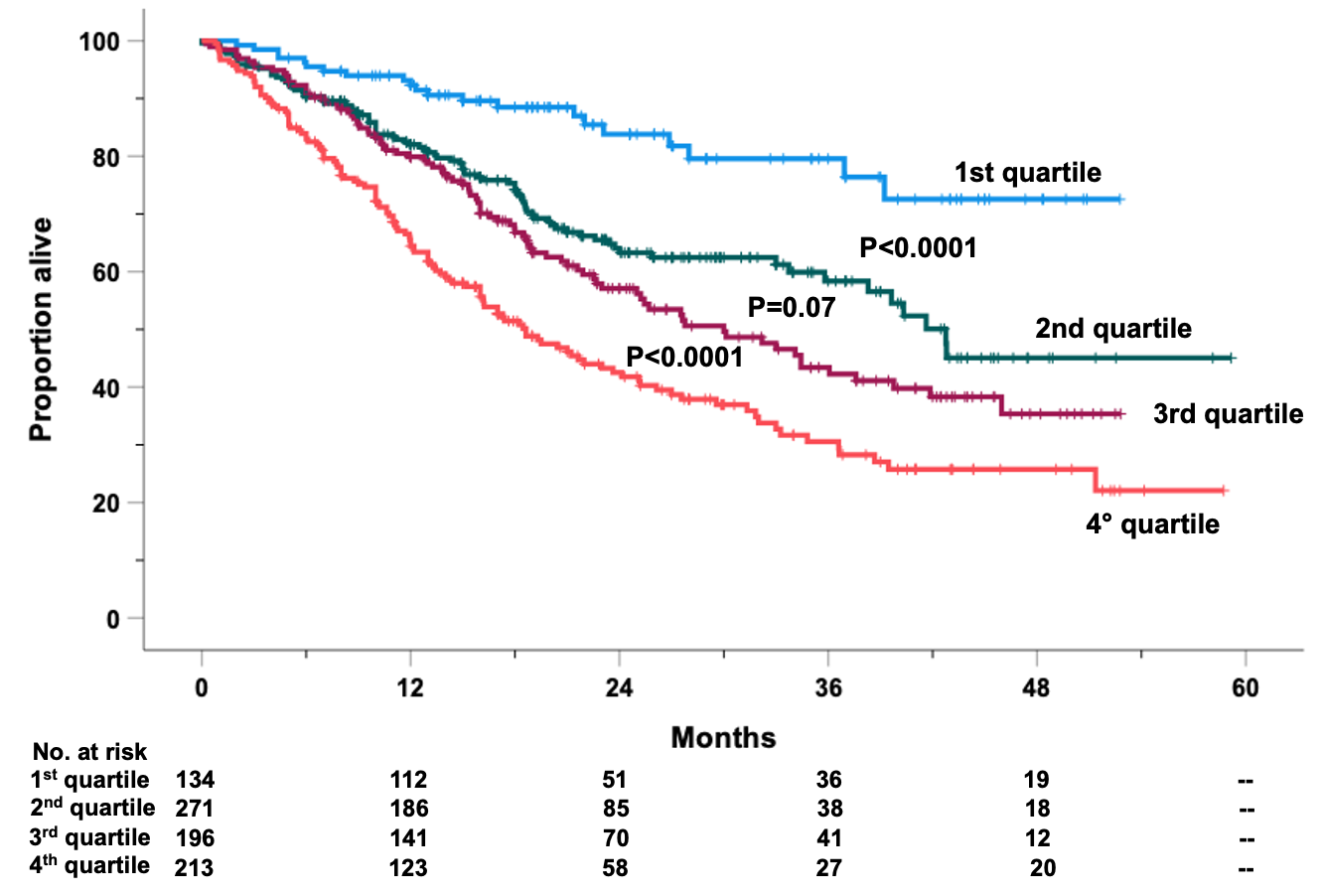


**Supplementary Figure 6.** Overall survival (OS) of the retrospective relapsed/refractory (RR) multiple myeloma cases treated with KRd or EloRd by SRS*^KRd/EloRd^*. The percentage weights were summed up on an individual basis, and cases clustered by quartiles. This analysis was carried out in 814 multiple myeloma cohort treated with KRd (559 cases) or EloRd (255 cases) in which all the 5 variables were available.


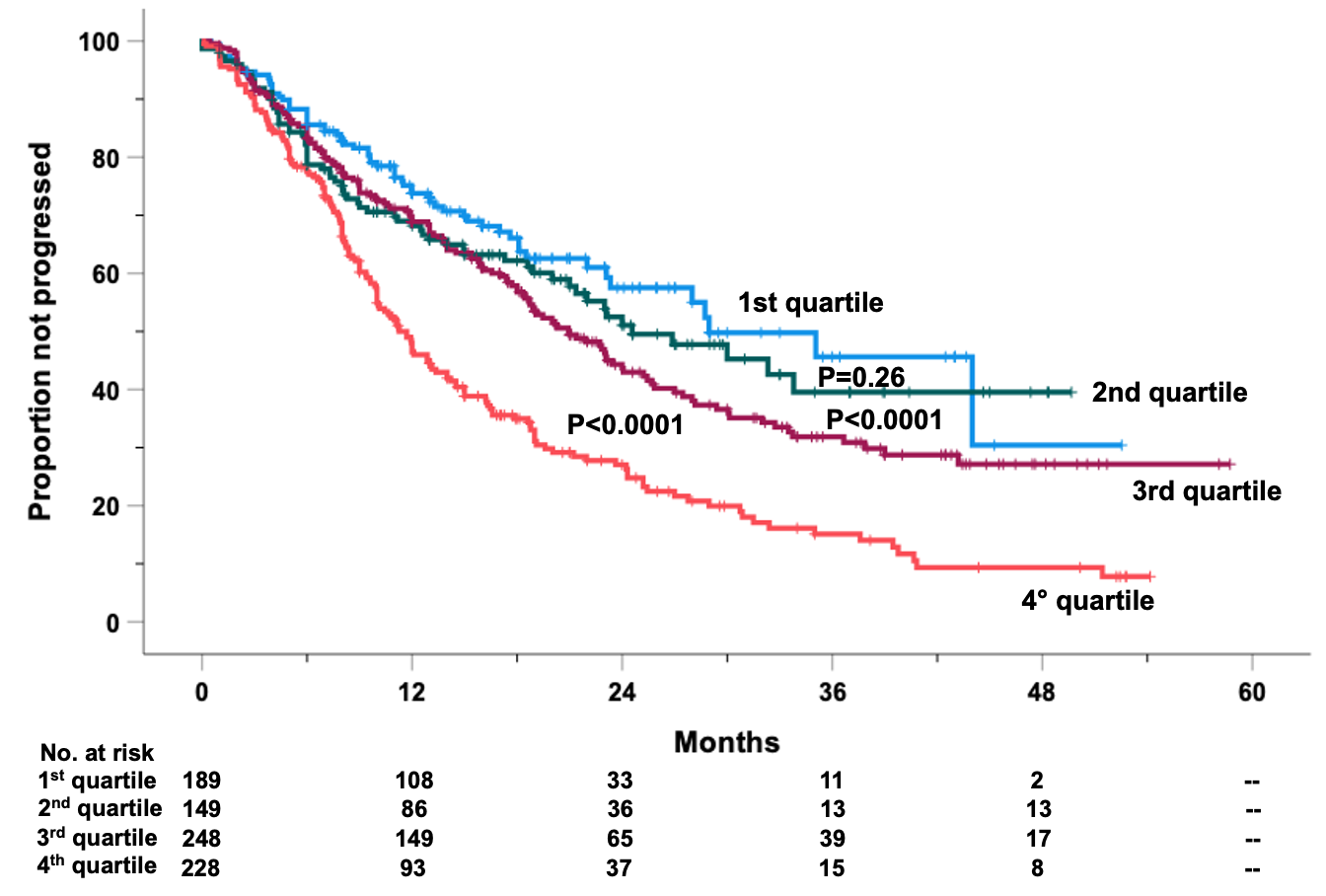


**Supplementary Figure 7.** Progression-free survival (PFS) of the retrospective relapsed/refractory (RR) multiple myeloma cases treated with KRd or EloRd by by PRS*^KRd/EloRd^.* The percentage weights were summed up on an individual basis, and cases clustered by quartiles. This analysis was carried out in 814 multiple myeloma cohort treated with KRd (559 cases) or EloRd (255 cases) in which all the 4 variables were available.


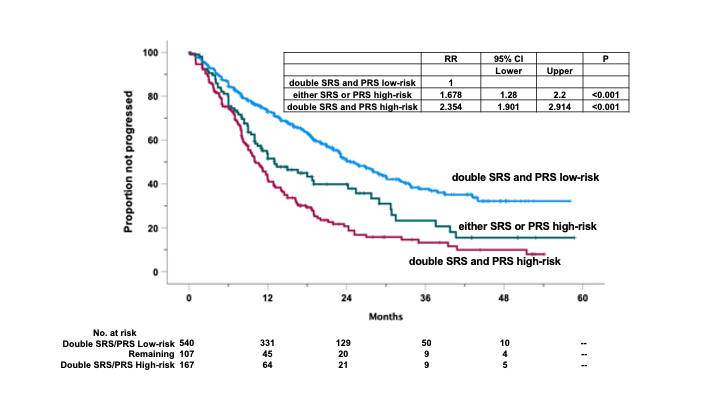


**Supplementary Figure 8**. Progression-free survival (PFS) of the retrospective relapsed/refractory (RR) multiple myeloma cases treated with KRd or EloRd clustered by a combination of progression-free (PRS) and overall survival (SRS) risk scores in double low- (i.e., low risk in both models) double high-risk (i.e., high-risk in both models) and either SRS or PRS high-risk.


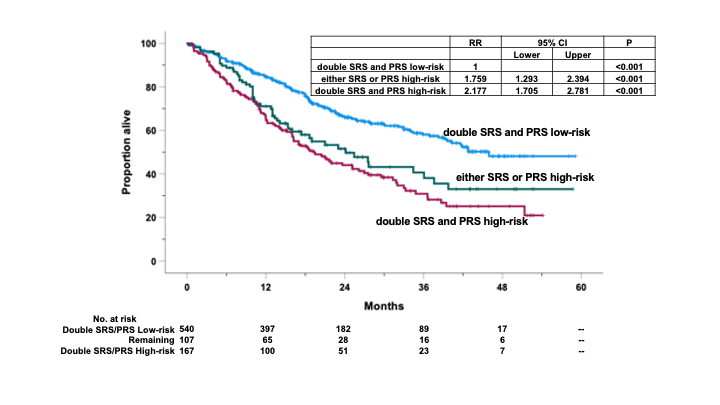


**Supplementary Figure 9**. Overall survival (OS) of the retrospective relapsed/refractory (RR) multiple myeloma cases treated with KRd or EloRd clustered by a combination of progression-free (PRS) and overall survival (SRS) risk scores in double low- (i.e., low risk in both models) double high-risk (i.e., high-risk in both models) and either SRS or PRS high-risk.
